# Genetically Diverse Coronaviruses in Wild Bird Populations of Northern England

**DOI:** 10.3201/eid1507.090067

**Published:** 2009-07

**Authors:** Laura A. Hughes, Carol Savage, Clive Naylor, Malcolm Bennett, Julian Chantrey, Richard Jones

**Affiliations:** University of Liverpool, Neston, United Kingdom

**Keywords:** Coronavirus, infectious bronchitis, IBV, wild birds, viruses, zoonoses, England, dispatch

## Abstract

Infectious bronchitis virus (IBV) causes a costly respiratory viral disease of chickens. The role of wild birds in the epidemiology of IBV is poorly understood. We detected diverse coronaviruses by PCR in wildfowl and wading birds in England. Sequence analysis showed some viruses to be related to IBV.

Infectious bronchitis virus (IBV), a group 3 coronavirus, causes a costly viral disease of chickens that is found worldwide (*1*). It can cause respiratory disease in chickens of all ages and a loss of production and egg quality in mature hens (*2*). Some strains are nephropathogenic, resulting in renal-induced mortality rates of up to 25% for susceptible flocks (*1*). Currently, the poultry industry controls disease through the use of vaccines. However, IBV continuously generates antigenic variants; current vaccines offer no protection against some of them (*3*). Wild birds may play a role as both reservoirs and long-distance vectors of IBV.

Recently, group 3 coronaviruses genetically similar to IBV were detected in healthy galliform and nongalliform birds (*4*,*5*). This finding may suggest that wild birds are able to carry IBV-like viruses asymptomatically. Other studies have detected coronaviruses that are genetically distinct from IBV in wild birds, including graylag geese (*Anser anser*), rock doves (*Columba livia*), mallards (*Anas platyrhynchos*), Chinese bulbuls (*Pycnonotus sinensis*), red-whiskered bulbuls (*Pycnonotus jocosus*), gray-backed thrushes (*Turdus hortulorum*), blackbirds (*Turdus merula*), white-rumped munias (*Lonchura striata*), and scaly-breasted munias (*Lonchura punctulata*) (*6*,*7*). We report the detection and characterization of group 3 coronaviruses, some of which appear to be related to IBV, from wild bird populations sampled in northern England.

## The Study

Serial cross-sectional surveys of wild bird populations throughout northern England were undertaken from July 2004 through January 2007. Samples were collected from 441 individual wild birds of 42 species, including both migratory and resident species (Table 1). Fecal samples and oropharyngeal swabs were collected from live birds that had been caught primarily for ringing purposes (*8*) and from dead wildfowl and corvids provided by licensed shooters. Biometric data and information concerning the location where birds had been trapped or shot were recorded (*8*). Samples were placed in virus transport media (Eagle minimum essential medium supplemented with 10% fetal calf serum, penicillin, and streptomycin [10,000 units penicillin, 10 µg streptomycin/mL] and amphotericin B [250 µg/mL]); samples collected from the same wild bird species or genus were pooled in groups of 5 and frozen at –80°C until required.

**Table 1:** Wild bird species from which oropharyngeal swabs and fecal samples were collected and screened for coronavirus RNA, England

Taxonomic family	Common name	Latin name	No. birds screened
Sulidae	Northern gannet	*Morus bassanus*	1
Phalacrocoracidae	Great cormorant	*Phalacrocorax carbo*	2
Ardeidae	Grey heron	*Ardea cinerea*	35
Anatidae	Whooper swan	*Cygnus cygnus*	55
	Mute swan	*Cygnus olor*	25
	Pink-footed goose	*Anser brachyrhynchus*	3
	Greylag goose	*Anser anser*	1
	Canada goose	*Branta canadensis*	1
	Brent goose	*Branta bernicla*	7
	Common shelduck	*Tadorna tadorna*	2
	Mallard	*Anas platyrhynchos*	34
	Northern pintail	*Anas acuta*	9
	Northern shoveler	*Anas clypeata*	1
	Eurasian wigeon	*Anas penelope*	33
	Common teal	*Anas crecca*	18
	Common pochard	*Aythya ferina*	10
Accipitridae	Eurasian sparrowhawk	*Accipiter nisus*	4
Falconidae	Common kestrel	*Falco tinnunculus*	1
Rallidae	Common moorhen	*Gallinula chloropus*	1
	Common coot	*Fulica atra*	13
Haematopodidae	Eurasian oystercatcher	*Haematopus ostralegus*	42
Charadriidae	Ringed plover	*Charadrius hiaticula*	5
Scolopacidae	Dunlin	*Calidris alpina*	8
	Sanderling	*Calidris alba*	8
	Red knot	*Calidris canutus*	14
	Ruddy turnstone	*Arenaria interpres*	26
	Common redshank	*Tringa totanus*	3
	Eurasian woodcock	*Scolopax rusticola*	3
Laridae	Black-headed gull	*Larus ridibundus*	7
	Herring gull	*Larus argentatus*	15
	Great black-backed gull	*Larus marinus*	1
	Lesser black-backed gull	*Larus fuscus*	2
Sternidae	Common tern	*Sterna hirundo*	25
Alcidae	Common guillemot	*Uria aalge*	1
Columbidae	Rock dove	*Columba livia*	2
	Eurasian collared dove	*Streptopelia decaocto*	5
Tytonidae	Barn owl	*Tyto alba*	4
Prunellidae	Dunnock/Hedge accentor	*Prunellla modularis*	1
Turdidae	Northern wheatear	*Oenanthe oenanthe*	2
Passeridae	House sparrow	*Passer domesticus*	4
Fringillidae	European greenfinch	*Carduelis chloris*	2
	Eurasian siskin	*Carduelis spinus*	5

Viral RNA was extracted from pooled fecal samples and pooled oropharyngeal swabs by using a QIAamp Viral RNA Mini Kit (QIAGEN, Crawley, UK) following the manufacturer’s instructions. Reverse transcription–PCR was used to detect avian coronaviruses as previously described (*9*). The primers UTR41+ (5′-ATGTCTATCGCCAGGGAAATGTC-3′) and UTR11- (5′-GCTCTAACTCTATACTAGCCTA-3′) targeted the 3′ untranslated region (UTR) of the coronavirus genome, which is highly conserved among all known types of IBV (*9*). This procedure was followed by use of a heminested PCR with the same forward primer but the reverse primer UTR hemi- (5′-CTTAAACTAAAATTTAGCTCTTCC-3′) under the same reaction conditions as the initial PCR, which had an expected product size of 214 bp. PCR products were purified by using a commercial purification kit (QIAquick PCR Purification Kit; QIAGEN) according to the manufacturer’s instructions and were sequenced commercially (Cogenics, Essex, UK) as recommended by the manufacturer (ABI 3730xl DNA Analyser; Applied Biosystems, Warrington, UK). Nucleotide sequences derived from this study have been deposited in the GenBank sequence database under accession nos. FJ490193–FJ490199.

Sequences were aligned with previously published coronavirus sequences obtained from GenBank by using the Clustal program within the MEGA 4.0 package (*10*) (Appendix Figure). Phylogenetic analyses were conducted in MEGA 4.0. Phylogenies were estimated by using a minimum-evolution method (*11*), and evolutionary distances were computed by using the Tamura-Nei method (*12*). Phylogenetic trees were drawn to scale; branch lengths in the same units as those of the evolutionary distances were used to infer the phylogenetic tree. Bootstrap analysis using 1,000 repetitions provided support for individual nodes (*13*).

Animal-level prevalences and confidence limits, based on pooled samples, were estimated by using a pooled prevalence calculator (www.ausvet.com.au/pprev). Generalized linear modeling was used to calculate maximum-likelihood estimates of prevalence and confidence limits (*14*).

Coronavirus RNA was detected in 7 fecal sample pools (Table 2), giving an individual animal–level prevalence estimate of 1.6% (95% confidence interval 0.7–3.1). Of the pools with positive results for coronavirus, 4 were collected from ducks (designated Anas/UK/p20/2005, Anas/UK/p33/2005, Anas/UK/p42/2005 and Anas/UK/p71/2005; Table 2). Another pool contained samples from whooper swans (*Cygnus cygnus*) (whooper swan/UK/p3/2005), 1 consisted of samples from red knots (*Calidris canutus*) (red knot/UK/p60/2006), and 1 contained samples from Eurasian oystercatchers (*Haematopus ostralegus*) (oystercatcher/UK/p17/2006). PCR-positive pools were from birds sampled in estuarine, salt marsh, or standing water habitats (Table 2). All birds appeared to be healthy. All pooled oropharyngeal samples were PCR negative.

**Table 2:** Characteristics of wild birds that contributed to CoV RT-PCR–positive fecal pools*

Pool	Source species	Habitat†	Age	Sex	CoV detected
1	Whooper swan	Standing fresh water	Juvenile	M	Whooper swan/UK/p3/2005
	Whooper swan	Standing fresh water	Adult	M	
	Whooper swan	Standing fresh water	Adult	M	
	Whooper swan	Standing fresh water	Adult	M	
	Whooper swan	Standing fresh water	Adult	F	
2	Teal	Estuarine salt marsh	Juvenile	F	Anas/UK/p20/2005
	Mallard	Estuarine salt marsh	Unknown	M	
	Mallard	Estuarine salt marsh	Unknown	M	
	Mallard	Estuarine salt marsh	Unknown	M	
	Wigeon	Estuarine salt marsh	Juvenile	F	
3	Pintail	Estuarine salt marsh	Unknown	F	Anas/UK/p33/2005
	Pintail	Estuarine salt marsh	Unknown	M	
	Wigeon	Estuarine salt marsh	Unknown	F	
	Mallard	Estuarine salt marsh	Unknown	F	
	Mallard	Estuarine salt marsh	Unknown	F	
4	Pintail	Estuarine salt marsh	Unknown	F	Anas/UK/p71/2005
	Wigeon	Estuarine salt marsh	Unknown	M	
	Pintail	Estuarine salt marsh	Unknown	M	
	Wigeon	Estuarine salt marsh	Unknown	M	
	Wigeon	Estuarine salt marsh	Unknown	M	
5	Red knot	Estuarine	Unknown	Unknown	Red knot/UK/p60/2006
	Red knot	Estuarine	Unknown	Unknown	
	Red knot	Estuarine	Unknown	Unknown	
	Red knot	Estuarine	Unknown	Unknown	
	Red knot	Estuarine	Unknown	Unknown	
6	Oystercatcher	Estuarine	Adult	Unknown	Oystercatcher/UK/p17/2006
	Oystercatcher	Estuarine	Juvenile	Unknown	
	Oystercatcher	Estuarine	Adult	Unknown	
	Oystercatcher	Estuarine	Adult	Unknown	
	Oystercatcher	Estuarine	Adult	Unknown	
7	Wigeon	Estuarine salt marsh	Unknown	F	Anas/UK/p42/2005
	Wigeon	Estuarine salt marsh	Unknown	F	
	Wigeon	Estuarine salt marsh	Unknown	F	
	Wigeon	Estuarine salt marsh	Unknown	F	
	Wigeon	Estuarine salt marsh	Unknown	M	

*Cov, coronavirus; RT-PCR, reverse transcription–PCR. †Habitat type in which wild bird was caught for sampling.

Phylogenetic analyses were based on a final usable sequence of 146 nt after removal of primer sites (Appendix Figure). Nucleotide distances between coronavirus sequences derived from this study were 0.0%–15.6%. Sequences detected in 3 pooled duck samples and a sequence derived from a pool of whooper swan samples clustered with sequence from an IBV H120 (Massachusetts) vaccine strain. Sequences within this cluster were relatively homogenous with low within-group distance values (0.0%–2.8%). Within this cluster, bootstrap support for the individual nodes was relatively low (Figure).

**Figure:**
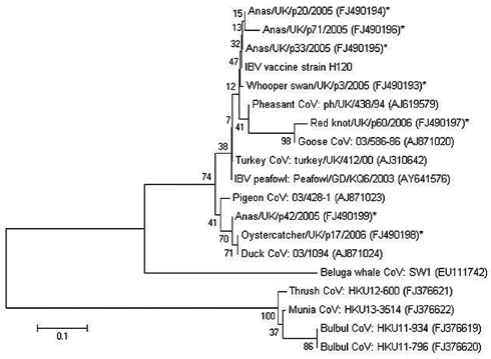
Minimum-evolution tree (*11*) of coronaviruses based on a 146-bp fragment of the 3′ untranslated region of infectious bronchitis virus (IBV). Evolutionary distances were computed by using the Tamura-Nei method (*12*) and are in the units of the number of base substitutions per site. Coronaviruses detected in wild birds by this study are denoted with an asterisk. Previously published coronavirus sequences from different sources were included for comparative purposes. GenBank accession numbers are shown in brackets. The percentage of replicate trees in which the associated taxa clustered together in the bootstrap test (1,000 replicates) is shown next to the branches (*13*). The tree is drawn to scale; branch lengths are in the same units as those of the evolutionary distances used to infer the phylogenetic tree. Phylogenetic analyses were conducted in MEGA4 (*10*). CoV, coronavirus. Scale bar indicates nucleotide substitutions per site.

Coronavirus sequences detected in red knots clustered with a previously described goose coronavirus; divergence at the nucleotide level was 2.0%. Sequences from viruses detected in samples from Eurasian oystercatchers and ducks clustered with sequence from a published duck coronavirus (*7*). The sequence from Eurasian oystercatchers was identical to that of the published duck coronavirus; distance values within this cluster were 0.0%–1.0%.

## Conclusions

Although samples were collected from wild bird populations comprising 46 species of wild birds from numerous and diverse habitats, coronavirus RNA was detected only in wildfowl (Anseriforms) and waders (Charadriiformes). Coronaviruses have been detected previously in wildfowl species, rock doves, wild peafowl, and some passerine species (*4*–*7*). All wild birds from which coronaviruses were detected in this study appeared to be healthy. Although IBV is recognized primarily as a respiratory agent, it has been demonstrated that certain strains are able to replicate in the chicken intestine without obvious clinical disease (*15*).

Phylogenetic analysis showed that coronavirus sequences detected by this study were genetically diverse. Virus sequences from 3 pools of fecal samples from ducks and whooper swans shared high nucleotide sequence identity with sequence from the IBV H120 vaccine strain, which is commonly used for the vaccination of commercial chickens worldwide. Coronaviruses sharing a high degree of identity with the IBV H120 vaccine strain have been detected previously in healthy, unvaccinated, domestic peafowl and as well as wild peafowl in China (*4*,*5*). These viruses may be revertant attenuated vaccine strains that have arisen as a result of the widespread use of IBV vaccines in the local poultry population in China. To understand their potential role as reservoirs of IBV strains, further surveillance for coronaviruses in wild bird populations is needed. It would be useful to determine the number and genome position of accessory genes of the coronaviruses detected in wild birds and to compare them with those of IBV. More detailed genetic characterization of the viruses detected including, for example, the S1 spike gene, is also needed. The detection of coronaviruses that appear to be related to IBV in wild migratory birds raises interesting questions as to their role in the transmission, dissemination, and evolution of IBV strains.

## Supplementary Material

Appendix Figure Multiple-sequence alignment of a fragment of the 3? untranslated region of coronaviruses detected in wild birds in this study and other previously published group 3 coronavirus sequences from wild birds and a beluga whale. Viruses detected by this study are marked with an asterisk. GenBank accession numbers for all sequences are shown in parentheses. Identical nucleotides are marked with a period (.). Sequences were aligned using the Clustal program within the MEGA 4.0 software package (10).

## References

[1] Cavanagh D, Gelb J. Infectious bronchitis. In: Saif YM, editor. Diseases of poultry, 12th ed. Ames (IA): Wiley-Blackwell Publishing; 2008. p. 101–20.

[2] Worthington KJ, Currie RJ, Jones RC. A reverse transcriptase–polymerase chain reaction survey of infectious bronchitis virus genotypes in Western Europe from 2002 to 2006. Avian Pathol. 2008;37:247–57. 10.1080/03079450801986529 18568650

[3] Adzhar A, Gough RE, Haydon D, Shaw K, Britton P, Cavanagh D. Molecular analysis of the 793/B serotype of infectious bronchitis virus in Great Britain. Avian Pathol. 1997;26:625–40. 10.1080/03079459708419239 18483932

[4] Liu S, Chen J, Chen J, Kong X, Shao Y, Han Z, Isolation of avian infectious bronchitis coronavirus from domestic peafowl (*Pavo cristatus*) and teal (*Anas*). J Gen Virol. 2005;86:719–25. 10.1099/vir.0.80546-0 15722532

[5] Sun L, Zhang GH, Jiang JW, Fu JD, Ren T, Cao WS, A Massachusetts prototype-like coronavirus isolated from wild peafowls is pathogenic to chickens. Virus Res. 2007;130:121–8. 10.1016/j.virusres.2007.06.003 17629993 PMC7114154

[6] Woo PC, Lau SK, Lam CS, Lai KK, Huang Y, Lee P, Comparative analysis of complete genome sequences of three avian coronaviruses reveals a novel group 3c coronavirus. J Virol. 2008;83:908–17. 10.1128/JVI.01977-08 18971277 PMC2612373

[7] Jonassen CM, Kofstad T, Larsen IL, Lovland A, Handeland K, Follestad A, Molecular identification and characterization of novel coronaviruses infecting graylag geese (*Anser anser*), feral pigeons (*Columbia livia*) and mallards (*Anas platyrhynchos*). J Gen Virol. 2005;86:1597–607. 10.1099/vir.0.80927-0 15914837

[8] Redfern CPE, Clark JA. Ringer’s manual. Thetford (UK): British Trust for Ornithology; 2001.

[9] Cavanagh D, Mawditt K, Sharma M, Drury SE, Ainsworth HL, Britton P, Detection of a coronavirus from turkey poults in Europe genetically related to infectious bronchitis virus of chickens. Avian Pathol. 2001;30:355–68. 10.1080/03079450120066368 19184921

[10] Tamura K, Dudley J, Nei M, Kumar S. MEGA4: Molecular evolutionary genetics analysis (MEGA) software version 4.0. Mol Biol Evol. 2007;24:1596–9. 10.1093/molbev/msm092 17488738

[11] Rzhetsky A, Nei M. A simple method for estimating and testing minimum evolution trees. Mol Biol Evol. 1992;9:945–67.

[12] Tamura K, Nei M. Estimation of the number of nucleotide substitutions in the control region of mitochondrial DNA in humans and chimpanzees. Mol Biol Evol. 1993;10:512–26. 8336541 10.1093/oxfordjournals.molbev.a040023

[13] Felsenstein J. Confidence limits on phylogenies: an approach using the bootstrap. Evolution. 1985;39:783–91. 10.2307/2408678 28561359

[14] Williams CJ, Moffitt CM. A critique of methods of sampling and reporting pathogens in populations of fish. J Aquat Anim Health. 2001;13:300–9. 10.1577/1548-8667(2001)013<0300:ACOMOS>2.0.CO;2

[15] Ambali AG, Jones RC. Early pathogenesis in chicks of infection with an enterotropic strain of infectious bronchitis virus. Avian Dis. 1990;34:809–17. 10.2307/1591367 2177973

