# Supplementary figures and images for: Genetically Diverse Coronaviruses in Wild Bird Populations of Northern England
Source: Emerg Infect Dis. 2009 Jul;15(7):1091–4. doi: 10.3201/eid1507.090067 (PMC2744231; doi:10.3201/eid1507.090067)

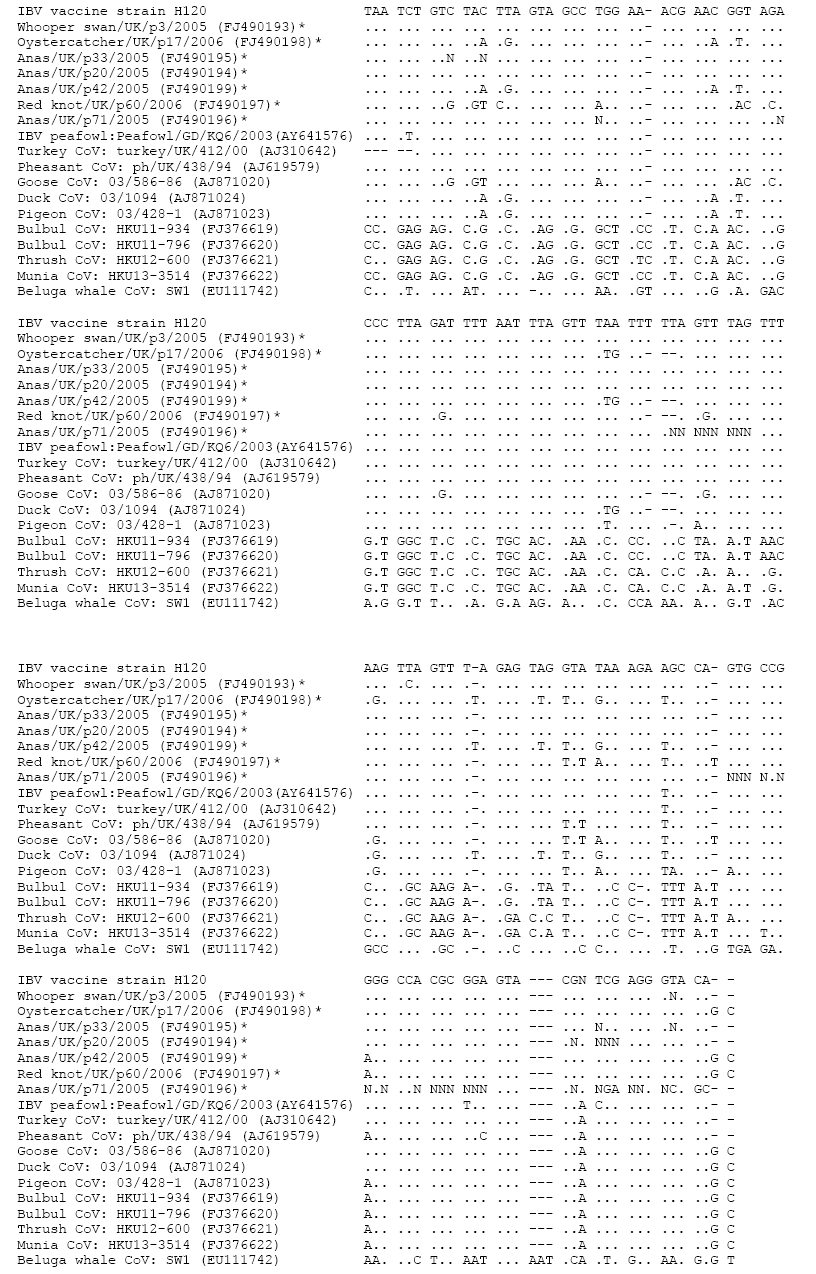

Supplement: Appendix Figure — Multiple-sequence alignment of a fragment of the 3? untranslated region of coronaviruses detected in wild birds in this study and other previously published group 3 coronavirus sequences from wild birds and a beluga whale. Viruses detected by this study are marked with an asterisk. GenBank accession numbers for all sequences are shown in parentheses. Identical nucleotides are marked with a period (.). Sequences were aligned using the Clustal program within the MEGA 4.0 software package (10). [file 09-0067_appF-s1.gif]
